# The clinical relevance of childhood manic symptoms

**DOI:** 10.1192/j.eurpsy.2021.122

**Published:** 2021-08-13

**Authors:** S. Frangou

**Affiliations:** Pychiatry, Icahn School of Medicine at Mount Sinai, New York City, United States of America

**Keywords:** childhood mania

## Abstract

**Background:**

The Adolescent Brain and Cognitive Development (ABCD) study, a US population-based sample of 10 year-olds, offers a unique opportunity to examine the neural correlates of manic-like symptoms presenting in children about to enter adolescence.

**Methods:**

The study will avail of the rich dataset of over 11,000 children aged 9-10 years at enrolment using data from the baseline and 2-year follow-up assessment. The analyses aim to track the evolution of manic-like symptoms between the two follow-up waves and test their sensitivity of their association with brain correlates.

**Results:**

Data analyses are ongoing and will focus on changes in manic-like symptoms, focusing on youth with remitting, persistent and emerging symptoms and examine their associations with brain structure and resting-state functional connectivity.

**Conclusions:**

The results will inform about the early trajectory of manic-like symptoms and offer new insights into their brain-related correlates.

**Disclosure:**

No significant relationships.

